# Prognostic factors and outcomes in Japanese lung transplant candidates with interstitial lung disease

**DOI:** 10.1371/journal.pone.0183171

**Published:** 2017-08-11

**Authors:** Kohei Ikezoe, Tomohiro Handa, Kiminobu Tanizawa, Toyofumi F. Chen-Yoshikawa, Takeshi Kubo, Akihiro Aoyama, Hideki Motoyama, Kyoko Hijiya, Shinsaku Tokuda, Yoshinari Nakatsuka, Yuko Yamamoto, Ayako Oshima, Shin-ichi Harashima, Sonoko Nagai, Toyohiro Hirai, Hiroshi Date, Kazuo Chin

**Affiliations:** 1 Department of Respiratory Medicine, Kyoto University Graduate School of Medicine, Kyoto, Japan; 2 Department of Respiratory Care and Sleep Control Medicine, Kyoto University Graduate School of Medicine, Kyoto, Japan; 3 Department of Thoracic surgery, Kyoto University Graduate School of Medicine, Kyoto, Japan; 4 Department of Diagnostic Imaging and Nuclear Medicine, Kyoto University Graduate School of Medicine, Kyoto, Japan; 5 Department of Metabolism and Clinical Nutrition, Kyoto University Hospital, Kyoto, Japan; 6 Department of Diabetes, Endocrinology, and Nutrition, Kyoto University Graduate School of Medicine, Kyoto, Japan; 7 Kyoto Central Clinic/Clinical Research Center, Kyoto, Japan; Stanford University, UNITED STATES

## Abstract

**Objective:**

Young patients with advanced interstitial lung disease (ILD) are potential candidates for cadaveric lung transplantation. This study aimed to examine clinical features, outcomes, and prognostic factors in Japanese ILD patients awaiting lung transplantation.

**Methods:**

We investigated the clinical features and outcomes of 77 consecutive candidates with ILD who were referred to Kyoto University Hospital and subsequently actively listed for lung transplant in the Japan Organ Transplant Network between 2010 and 2014.

**Results:**

Of the 77 candidates, 33 had idiopathic pulmonary fibrosis (IPF) and 15 had unclassifiable ILD. During the observational period, 23 patients (30%) received lung transplantations and 49 patients (64%) died before transplantation. Of the 33 patients with IPF, 13 (39%) had a family history of ILD and 13 (39%) had an “inconsistent with usual interstitial pneumonia pattern” on high-resolution computed tomography (HRCT). The median survival time from registration was 16.7 months, and mortality was similar among patients with IPF, unclassifiable ILD, and other ILDs. Using a multivariate stepwise Cox proportional hazards model, 6-min walking distance was shown to be an independent prognostic factor in candidates with ILD (per 10 m, hazard ratio (HR): 0.97; 95% confidence interval (CI): 0.95–0.99, p<0.01), while lower body mass index (HR: 0.83; 95% CI: 0.72–0.95, p < 0.01) independently contributed to mortality in patients with IPF.

**Conclusions:**

Japanese patients with ILD awaiting transplantation had very poor outcomes regardless of their specific diagnosis. A substantial percentage of IPF patients had an atypical HRCT pattern. 6-min walking distance in ILD patients and body mass index in IPF patients were independent predictors of mortality.

## Introduction

Interstitial lung disease (ILD) is difficult to cure and its prognosis is often poor. In particular, idiopathic pulmonary fibrosis (IPF) has very poor outcome with a median survival of 2–3 years from the time of diagnosis [1]. Meanwhile, patients with other ILDs, such as idiopathic nonspecific interstitial pneumonia (NSIP), connective tissue disease-associated ILD (CTD-ILD), and chronic hypersensitivity pneumonitis (CHP), sometimes have progressive disease, although their prognosis is usually more favorable than that of IPF [2–4]. Moreover, a substantial number of patients with ILD cannot be definitively classified as a specific ILD subtype, and are assigned as “unclassifiable ILD” [5]. A previous study showed that unclassifiable ILD has a prognosis that is intermediate between IPF and non-IPF ILDs [6].

Lung transplantation is the only effective therapy in patients with advanced ILD refractory to medical treatment. In Japan, cadaveric lung transplantation candidates must be younger than 60 years old at the time of registration on the Japan Organ Transplant Network (JOTN) waiting list because of a severe donor shortage [7]. As the number of cadaveric lung transplantation in Japan is limited, the average waiting time is more than 800 days [7, 8]. Moreover, lung allocation is performed based on accrued time on the waiting list, and there is no rule to prioritize patients with more rapidly progressing diseases such as IPF. Therefore, that Japanese lung transplant candidates with ILD may have poor outcome.

Hence, it is important to elucidate the prognostic factors and outcomes of lung transplant candidates with ILD in Japan. Recently, the simple scoring (GAP index), which consists of gender (G), age (A), and lung physiology variables (P), has been reported to predict mortality in patients with IPF [9]. However, prognostic factors of young (<60 years old) patients with ILD have not been elucidated.

We hypothesized that young lung transplant candidates with ILD have distinct clinical features and prognostic factors, and aimed to investigate the clinical features and outcomes in lung transplant candidates with ILD at our hospital.

## Materials and methods

### Study population

This study cohort comprised 77 consecutive lung transplant candidates with ILD who were referred to Kyoto University Hospital and subsequently actively listed for lung transplant in JOTN between April 2010 and July 2014. These candidates were selected based on the international guidelines [10]. IPF was diagnosed by surgical lung biopsy or high-resolution computed tomography (HRCT) based on the guidelines [1, 11]. Other idiopathic interstitial pneumonias (IIPs) were diagnosed as previously described [12–14], and CHP was diagnosed according to the established criteria [15]. We defined unclassifiable ILD as patients without a specific ILD diagnosis following multidisciplinary review of clinical, radiological, and pathological data [5]. This study was approved by the Ethics Committee of Kyoto University (E1355). Written informed consent was obtained from all subjects.

### Data collection

Clinical data were extracted from the lung transplantation registration database and medical records at our institute. Pulmonary function tests were performed using the CHESTAC system (Chest M.I. Inc., Tokyo, Japan), and diffusing capacity of the lung for carbon monoxide (DL_CO_) was determined using the single-breath technique. The GAP stage was defined using the method previously reported by Ley et al [9]. The 6-min walk test (6MWT) was conducted according to the American Thoracic Society guidelines [16]. Supplemental oxygen flow during 6MWT was recorded. Pulmonary hypertension was defined as mean pulmonary artery pressure ≥25 mmHg by right heart catheterization or by estimated systolic pulmonary artery pressure ≥40 mmHg by echocardiography in six patients without right heart catheterization data. The HRCT findings in 33 patients with IPF were classified as usual interstitial pneumonia (UIP) pattern, possible UIP pattern, or “inconsistent with UIP pattern” per the guidelines [1]. We observed patients from the date of registration at JOTN to the date of last contact, transplantation, or death.

### Statistical analysis

Statistical analyses were performed using JMP version 10 (SAS Institute Inc., Cary, NC, USA) and R version 3.3.1 (R Studio, Boston, MA, USA). Results of continuous variables are presented as mean ± standard deviation. Differences in continuous and categorical variables among the three groups were assessed by analysis of variance, followed by the Tukey-Kramer post-hoc test and the chi-square test, respectively. To identify factors predictive of mortality, we first used a conventional Cox proportional hazards model (treating lung transplants as censoring events). Next, we used Fine and Gray subdistribution hazards models while treating transplantation as a competing risk [17]. In the multivariate Cox proportional hazards model analysis, a stepwise variable-selecting procedure was performed, and parameters with p < 0.1 in univariate analysis were exclusively entered. The proportional hazards assumption was assessed using Schoenfeld residuals. Survival curves were generated using the Kaplan–Meier method, and survival rates between subgroups were compared using the log-rank test. All analyses were considered statistically significant for p < 0.05.

## Results

### Diagnoses and outcomes

The diagnoses and outcomes of 77 lung transplant candidates with ILD are shown in Table 1. Of these patients, 53 (69%) were diagnosed with IIP and 17 (22%) and 7 (9%) were diagnosed with CTD-ILD and CHP, respectively. Of the 53 patients with IIP, 33 had IPF and 15 of the 20 patients with non-IPF IIP were diagnosed with unclassifiable ILD. During the observation period (median: 14.4 months, range: 0.3–48.3 months), 23 patients (30%) received lung transplantations (cadaveric lung transplantation (n = 20) and living-donor lobar lung transplantation (n = 3)), while 49 patients (64%) died before transplantation. Among the remaining 5 patients, only one patient was still awaiting transplantation, while 4 patients were removed from our institutional list due to stable disease (n = 2), contraindications (n = 1), and change in institution (n = 1). Among the 23 patients who received lung transplantations, 90-day survival was 100% (23/23), and 1-year survival was 94% (16/17).

**Table 1:** Diagnoses and outcomes of lung transplantation candidates with interstitial lung disease (n = 77).

Diagnosis	
Idiopathic interstitial pneumonia	53 (69%)
Idiopathic pulmonary fibrosis	33 (43%)
Nonspecific interstitial pneumonia	4 (5%)
Pleuroparenchymal fibroelastosis	1 (1%)
Unclassifiable ILD	15 (19%)
Connective tissue disease-associated ILD	17 (22%)
Chronic hypersensitivity pneumonitis	7 (9%)
Outcomes	
Death	49 (64%)
Transplantation	23 (30%)
Awaiting	1 (1%)
Removal from the list	4 (5%)

Data are number of cases (percentage). ILD, interstitial lung disease.

### Clinical characteristics and physiologic/laboratory data

The clinical characteristics of 77 candidates with ILD are shown in Table 2. Mean age was 49.0 ± 9.0 years, 48 patients (62%) were male, and 53 patients (69%) had received long-term oxygen therapy. Patients were classified into three groups: IPF (n = 33), unclassifiable ILD (n = 15), and other ILDs (n = 29), which included idiopathic NSIP (n = 4), idiopathic pleuroparenchymal fibroelastosis (n = 1), CTD-ILD (n = 17), and CHP (n = 7). Patients with IPF had a higher mean age than those with other ILDs, as well as a higher rate of male gender compared with the other two groups. Notably, 13 patients (39%) with IPF had a family history of ILD, which was significantly more common than in patients with other ILDs.

**Table 2:** Characteristics/Physiologic and laboratory data of lung transplantation candidates with interstitial lung disease (n = 77).

	All (n = 77)	IPF (n = 33)	Unclassifiable ILD (n = 15)	Other ILDs (n = 29)
**Characteristics**				
Age, years	49.0 ± 9.0	52.7 ± 7.4	47.3 ± 10.4	45.7 ± 8.7*
Male gender	48 (62%)	28 (85%)	5 (33%)*	15 (52%)*
ever smoker	45 (59%)	24 (73%)	7 (47%)	14 (50%)
BMI, kg/m^2^	21.2 ± 4.2	22.4 ± 3.8	20.2 ± 4.1	20.3 ± 4.5
Family history of ILD	17 (22%)	13 (39%)	3 (20%)	1 (4%)*
Long term oxygen therapy	53 (69%)	19 (58%)	10 (67%)	24 (83%)
History of acute exacerbation	13 (17%)	4 (12%)	1 (7%)	8 (29%)
History of pneumothorax	22 (29%)	5 (15%)	5 (36%)	12 (41%)*
**Physiologic/laboratory data**				
%FVC, %†	48.5 ± 15.8	54.6 ± 14.3	40.7 ± 13.3*	45.2 ± 16.6
>75	4 (6%)	3 (9%)	0 (2%)	1 (4%)
50–75	28 (39%)	16 (50%)	4 (27%)	8 (33%)
<50	39 (55%)	13 (41%)	11 (73%)	15 (63%)
%DL_CO_, %‡	26.2 ± 11.6	29.6 ± 11.5	24.5 ± 11.6	20.5 ± 10.1*
>55	1 (1%)	1 (3%)	0 (0%)	0 (0%)
36–55	9 (13%)	7 (22%)	1 (6%)	1 (4%)
≤35	40 (56%)	20 (63%)	7 (47%)	13 (54%)
Could not perform	21 (30%)	4 (12%)	7 (47%)	10 (42%)
6MWD, m	343 ± 165	436 ± 125	309 ± 160*	254 ± 156*
≥250	53 (69%)	30 (91%)	8 (53%)	15 (52%)
<250	24 (31%)	3 (9%)	7 (47%)	14 (48%)
Oxygen supplementation at 6MWT	47 (61%)	16 (48%)	9 (60%)	22 (76%)
Oxygen flow at 6MWT, L/min	2.2 ± 2.4	1.4 ± 1.6	2.1 ± 1.9	3.2 ± 3.0*
GAP stage I/II/III†, n (%)	15/50/6 (21/70/8)	9/23/0 (28/72/0)	2/11/2 (13/73/13)	4/16/4 (17/67/17)
LDH, mg/dL	236 ± 61	234 ± 56	213 ± 42	248 ± 72
KL-6, U/mL	1633 ± 1458	1380 ± 823	1443 ± 1003	1956 ± 2075

Data are number of cases (percentage) or mean ± standard deviation. IPF, idiopathic pulmonary fibrosis; ILD, interstitial lung disease; BMI, body mass index; %FVC, percent predicted forced vital capacity; %DL_CO_, percent predicted diffusing capacity of the lung for carbon monoxide; 6MWD, 6-min walking distance; 6MWT, the 6-min walk test; GAP stage, gender-age-physiology stage; LDH, lactate dehydrogenase; KL-6, Krebs von den Lungen-6. Other ILDs: idiopathic nonspecific interstitial pneumonia (n = 4), pleuroparenchymal fibroelastosis (n = 1), connective tissue disease-associated ILD (n = 17), and chronic hypersensitivity pneumonitis (n = 7).

*p < 0.05 vs. IPF group.

†n = 71

‡n = 50.

In addition, we investigated the HRCT pattern of 33 candidates with IPF. Among them, 16 patients (49%) showed a UIP pattern on HRCT and 4 patients showed a possible UIP pattern. However, the remaining 13 (39%) patients had an “inconsistent with UIP pattern”, although surgical lung biopsies showed a UIP pattern.

Physiologic and laboratory data from candidates with ILD are shown in Table 2. Six patients could not undergo pulmonary function testing because of the existence of pneumothorax, but pulmonary function data were available in the remaining 71 patients. Of these patients, 39 (55%) had a forced vital capacity (FVC) of <50% of the predicted value. Most candidates had a DL_CO_ of <35% of the predicted value or were unable to perform the DL_CO_ test due to low vital capacity or difficulty holding their breath. Most patients were GAP stages I and II due to their young age despite their pulmonary functions being extremely poor. The pulmonary function in patients with IPF was relatively preserved compared to the other two groups, though the distribution of GAP stage was not significantly different between groups.

### Survival analysis

**Fig 1:**
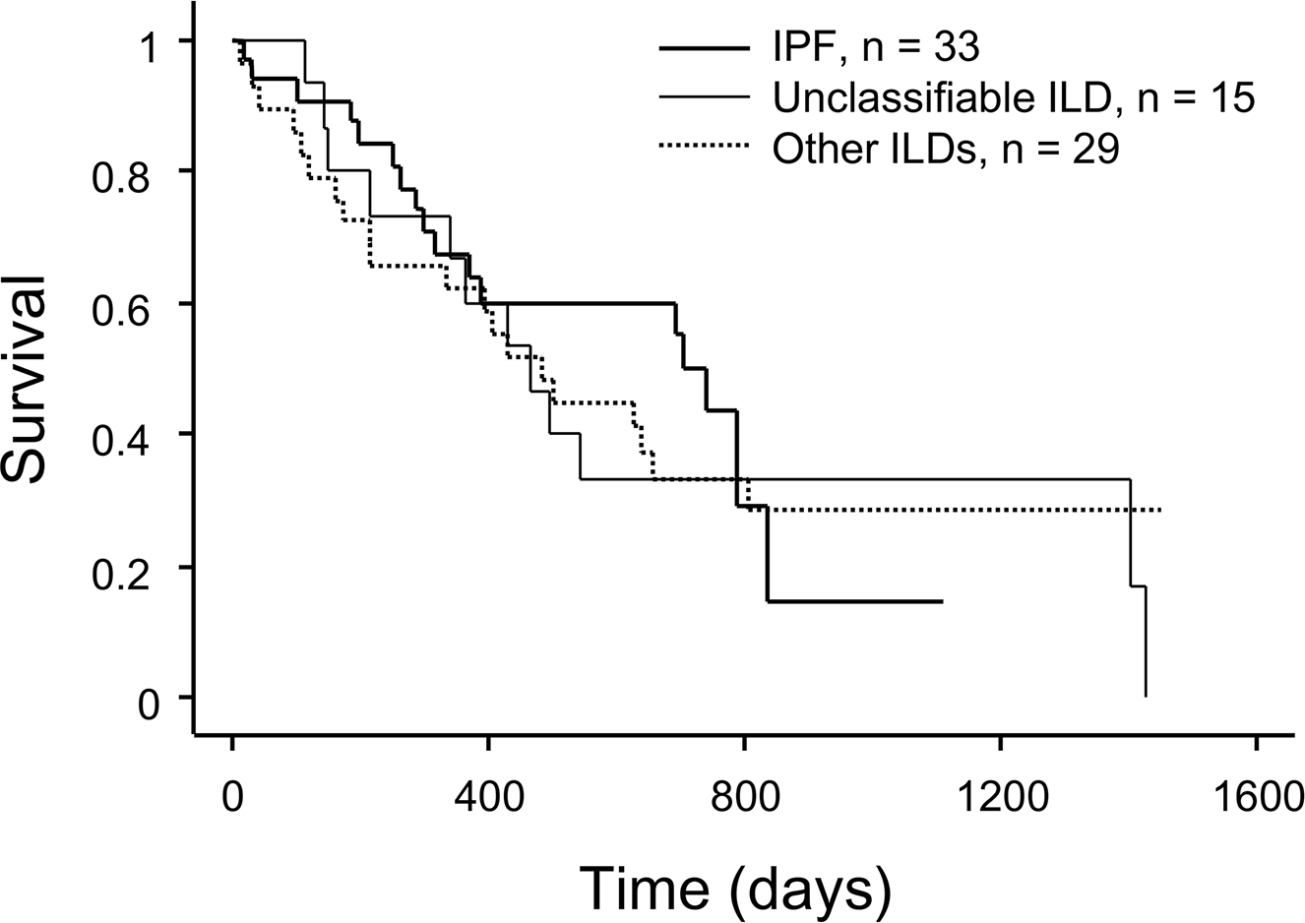
Kaplan−Meier survival curves for candidates with interstitial lung disease (ILD) grouped by diagnosis; idiopathic pulmonary fibrosis (n = 33), unclassifiable ILD (n = 15), and other ILDs (n = 29). Other ILDs: idiopathic nonspecific interstitial pneumonia (n = 4), pleuroparenchymal fibroelastosis (n = 1), connective tissue disease-associated ILD (n = 17), and chronic hypersensitivity pneumonitis (n = 7).

**Fig 2:**
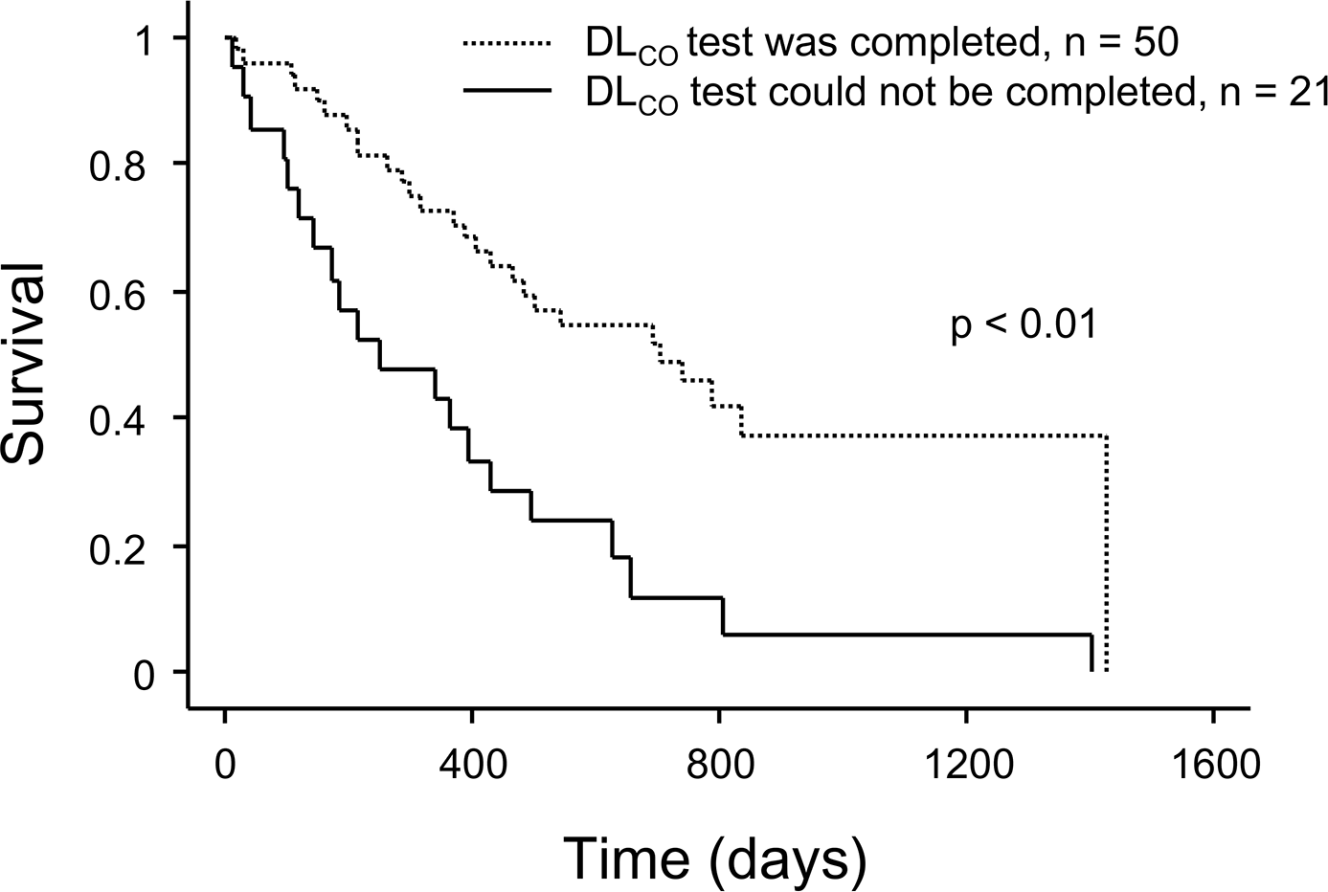
Kaplan−Meier survival curves for candidates with interstitial lung disease grouped based on the ability to perform the DL_CO_ test. p < 0.01 by log-rank test. DL_CO_, diffusing capacity of the lung for carbon monoxide.

Last, we performed survival analysis of the 77 candidates with ILD. The median survival time from registration to JOTN was 16.7 months. In a univariate Cox proportional hazards analysis, body mass index (BMI) (hazards ratio (HR) of 0.94 for each 1 kg/m^2^ increase in BMI; 95% confidence interval (CI): 0.88–0.996; p = 0.04), percent predicted FVC (%FVC) (HR: 0.78 for every 10% increase; 95% CI: 0.64–0.96, p = 0.02), percent predicted DL_CO_ (%DL_CO_) (HR: 0.60 for every 10% increase; 95% CI: 0.40–0.90, p = 0.01), the 6-min walking distance (6MWD) (HR: 0.97 for every 10 m increase; 95% CI: 0.95–0.99, p < 0.01), oxygen flow ≥2L/min at 6MWT (HR: 2.33; 95% CI: 1.28–4.25, p < 0.01), and GAP stage III (HR: 2.55; 95% CI: 1.07–6.04, p = 0.03) were significantly associated with mortality; however, DL_CO_ data were missing for 27 patients (Table 3). Kaplan–Meier survival curves showed that the mortality rate did not differ significantly among the three groups (Fig 1). We compared the mortality rate in patients whose DL_CO_ data were missing due to respiratory limitations (n = 21) and patients with available DL_CO_ data (n = 50), and showed that the patients without DL_CO_ data had significantly shorter survival times than the group with DL_CO_ data (p < 0.01, Fig 2).

**Table 3:** Cox proportional hazards model results for evaluating the risk of mortality in lung transplant candidates with interstitial lung disease (n = 77).

*Univariate analysis*	Hazards ratio	95%CI			p-value
Age, years	1.02	0.99	–	1.05	0.27
Male gender	0.82	0.46	–	1.46	0.49
IPF diagnosis	0.82	0.45	–	1.50	0.52
BMI, kg/m^2^	0.94	0.88	–	0.996	0.04
Ever smoker	0.93	0.52	–	1.66	0.81
Pulmonary hypertension	1.16	0.60	–	2.23	0.66
History of acute exacerbation	1.39	0.69	–	2.81	0.36
History of pneumothorax	1.26	0.69	–	2.31	0.45
%FVC, per 10%*	0.78	0.64	–	0.96	0.02
%DL_CO_, per 10%†	0.60	0.40	–	0.90	0.01
6MWD, per 10m	0.97	0.95	–	0.99	< 0.01
Oxygen flow ≥2L/min at 6MWT	2.33	1.28	–	4.25	< 0.01
GAP stage III	2.55	1.07	–	6.04	0.03
*Multivariate analysis*	Hazards ratio	95%CI			p-value
Model 1					
BMI, kg/m^2^	–		–		–
%FVC, per 10%	–		–		–
6MWD, per 10m	0.97	0.95	–	0.99	< 0.01
Oxygen flow ≥2L/min at 6MWT	–		–		–
*Model 2*					
BMI, kg/m^2^	–		–		–
6MWD, per 10m	0.97	0.95	–	0.99	< 0.01
Oxygen flow ≥2L/min at 6MWT	–		–		–
GAP stage III	–		–		–

CI, confidence interval; IPF, idiopathic pulmonary fibrosis; BMI, body mass index; ILD, interstitial lung disease; %FVC, percent predicted forced vital capacity; %DL_CO_, percent predicted diffusing capacity of the lung for carbon monoxide; 6MWD, 6-min walking distance; 6MWT, the 6-min walk test; GAP stage, gender-age-physiology stage.

*n = 71

†n = 50.

**Fig 3:**
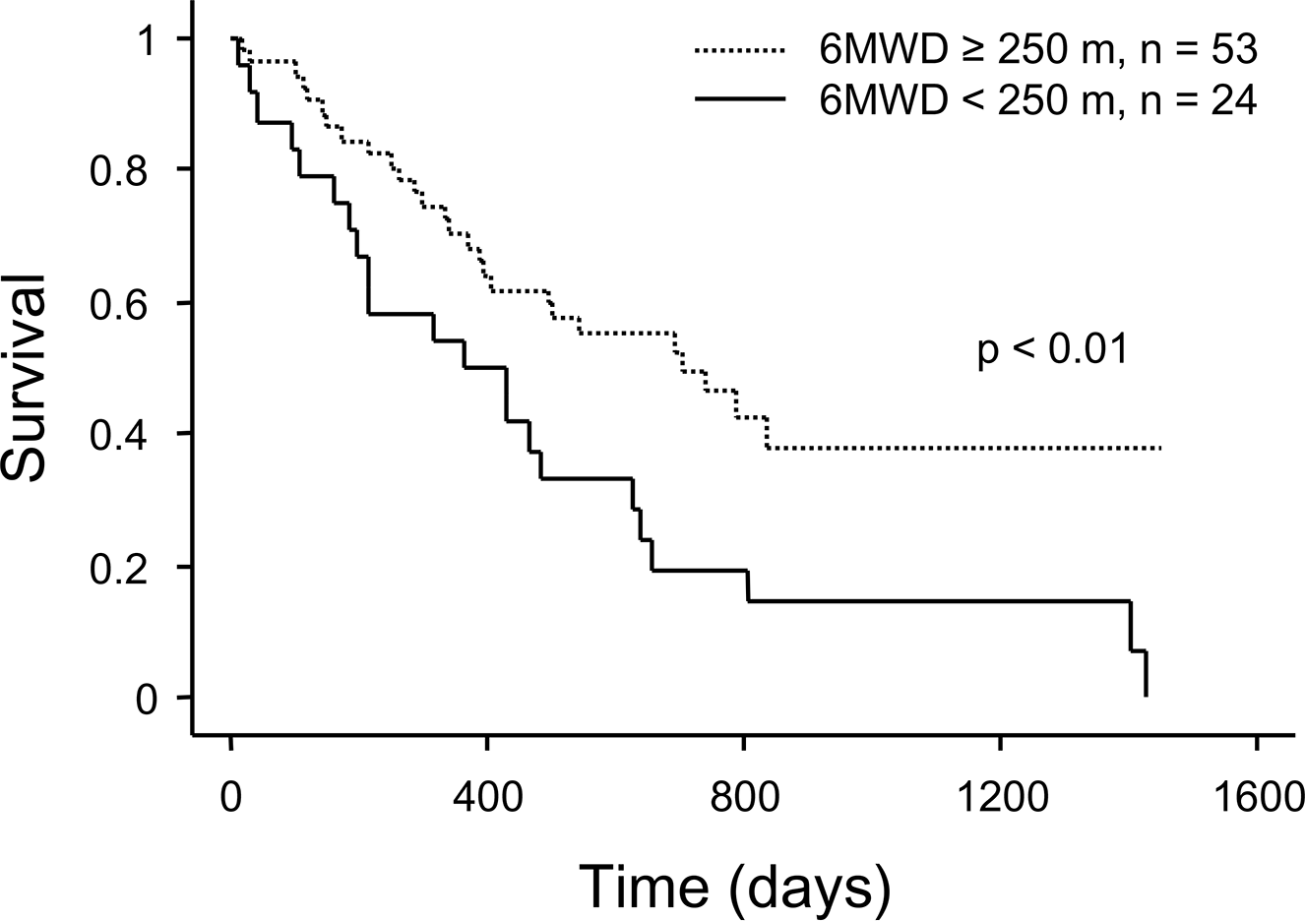
Kaplan−Meier survival curves for candidates with interstitial lung disease grouped based on 6MWD. p < 0.01 by log-rank test. 6MWD, 6-min walking distance.

In multivariate stepwise analyses, we excluded %DL_CO_ as a variable due to the considerable number of patients with missing data. We demonstrated that 6MWD independently contributed to mortality when selecting BMI, %FVC, 6MWD, and oxygen flow ≥2L/min at 6MWT (HR: 0.97 for every 10 m increase; 95% CI: 0.95–0.99, p < 0.01; model 1), or BMI, GAP stage III, 6MWD, and oxygen flow ≥2L/min at 6MWT (HR: 0.97 for every 10 m increase; 95% CI: 0.95–0.99, p < 0.01; model 2; Table 3). Validity of the proportional hazards assumption was confirmed using Schoenfeld residuals in multivariate analyses. To address the possible bias arising from censoring due to transplantation, we also performed Fine and Gray subdistribution hazards models while treating transplantation as a competing risk. In multiple stepwise analyses, 6MWD also independently contributed to mortality (HR: 0.97 for each 10 m increase; 95% CI: 0.95–0.99, p < 0.01, S1 Table). The cut-off value for 6MWD was set at 250 m based on the international consensus report [18]. Kaplan–Meier survival curves showed that patients with a 6MWD less than 250 m had significantly shorter survival times than those with a 6MWD ≥ 250 m (p < 0.01, Fig 3). Among the 24 patients with a 6MWD < 250 m, 22 (92%) died before transplantation, while 22 patients (42%) in the group with a 6MWD ≥ 250 m (n = 53) received a transplant.

**Fig 4:**
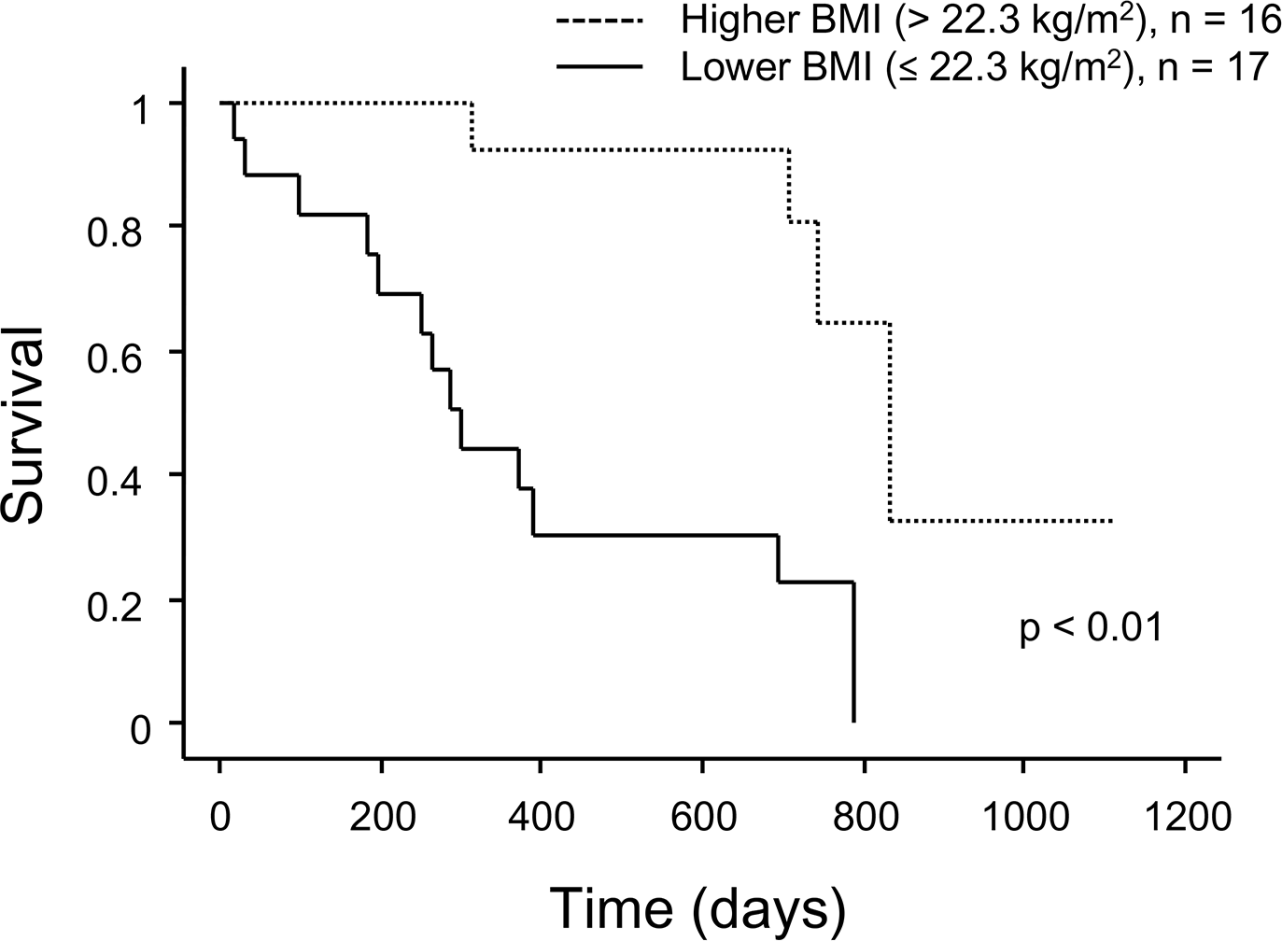
Kaplan−Meier survival curves for candidates with idiopathic pulmonary fibrosis grouped based on BMI. p < 0.01 by log-rank test. BMI, body mass index.

Additionally, survival analysis was performed in 33 candidates with IPF. Using the stepwise multivariate Cox proportional hazards model, BMI (HR: 0.83; 95% CI: 0.72–0.95, p < 0.01) and a history of acute exacerbation (AE) (HR: 4.50; 95% CI: 1.15–17.66, p = 0.03) independently contributed to increased mortality (Table 4). A univariate Fine and Gray subdistribution hazards model showed that a history of AE was not significantly associated with mortality. However, BMI also independently contributed to mortality in multiple stepwise Fine and Gray subdistribution hazards models (HR: 0.83; 95% CI: 0.75–0.91, p < 0.01, S2 Table). When patients with IPF were classified based on the median BMI value (22.3 kg/m^2^), patients with a lower BMI had significantly shorter survival times than those with a higher BMI (p < 0.01, Fig 4). In the lower BMI subgroup (n = 17), 13 (76%) patients died before transplantation and only 4 (24%) patients received a transplant, while 11 (69%) patients received a transplant in the higher BMI group (n = 16).

**Table 4:** Cox proportional hazards model results for evaluating the risk of mortality in patients with idiopathic pulmonary fibrosis (n = 33).

*Univariate analysis*	Hazards ratio	95%CI			p-value
Age, years	1.01	0.95	–	1.07	0.75
Male gender	0.38	0.12	–	1.20	0.099
BMI, kg/m^2^	0.85	0.74	–	0.97	0.01
Ever smoker	0.96	0.34	–	2.68	0.94
Pulmonary hypertension	0.91	0.20	–	4.06	0.90
History of acute exacerbation	3.38	0.91	–	12.58	0.07
History of pneumothorax	1.94	0.61	–	6.15	0.26
%FVC, per 10%*	0.78	0.57	–	1.08	0.14
%DL_CO_, per 10%†	0.48	0.27	–	0.84	0.01
6MWD, per 10m	0.96	0.93	–	1.00	0.08
Oxygen flow ≥2L/min at 6MWT	2.13	0.79	–	5.77	0.14
GAP stage II or III	2.70	0.76	–	9.66	0.13
*Multivariate analysis*	Hazards ratio	95%CI			p-value
Male gender	–		–		–
BMI, kg/m^2^	0.83	0.72	–	0.95	< 0.01
History of acute exacerbation	4.50	1.15	–	17.66	0.03
6MWD, m	–		–		–

CI, confidence interval; BMI, body mass index; ILD, interstitial lung disease; %FVC, percent predicted forced vital capacity; %DL_CO_, percent predicted diffusing capacity of the lung for carbon monoxide; 6MWD, 6-min walking distance; 6MWT, the 6-min walk test; GAP stage, gender-age-physiology stage.

*n = 32

†n = 28.

## Discussion

In the present study, we showed that 30% of lung transplant candidates with ILD received lung transplantation, while 64% died before transplantation in Japan. Among all candidates with ILD, 6MWD was independently associated with survival, while BMI was independently associated with survival in 33 patients with IPF. To the best of our knowledge, this is the first study to present prognostic factors in Japanese lung transplantation candidates with ILD.

Our study demonstrated two important findings regarding the prognosis for lung transplant candidates with ILD in Japan. First, these patients had very poor prognosis and the mortalities were similar among patients with IPF, unclassifiable ILD, and other ILDs. Our study results were different from previous reports, partly due to the lower frequency of IPF patients with severely impaired pulmonary function or exercise capacity compared with unclassifiable ILD and other ILDs at the time of registration (Table 2).

Second, 6MWD was independently associated with survival. The link between 6MWD and survival has been reported previously in patients with IPF and ILD [19–21]. 6MWT is a practical and simple test that requires no exercise equipment or advanced training for technicians [16]. In our study, 6MWT was performed by all participants, though most had severe pulmonary impairment, while the pulmonary function test was not performed by patients with complicated pneumothorax. Additionally, 6MWT is a reliable and reproducible test [22]. We set the cut-off value at 250 m for 6MWD based on the international consensus report [18], and showed that most of patients with a 6MWD of less than 250 m died before transplantation. Although a 6MWD below 250 m is one of the factors to consider for lung transplants in ILD patients [18], surviving until transplantation under the current time-based allocation system in Japan may be a challenge for these patients.

We considered that %DL_CO_ was an inappropriate predictive factor for prognosis since DL_CO_ data were unattainable in approximately a quarter of the participants. However, patients who could not complete the DL_CO_ test had significantly worse outcome than those who completed the DL_CO_ test, suggesting that DL_CO_ is a prognostic factor in patients with ILD [23–26]. It is, however, a major problem that the DL_CO_ test cannot be performed according to the standard approach when patients have an FVC of less than 1 L or cannot hold their breath for 10 s due to respiratory limitations [27]. 6MWD was strongly correlated with %DL_CO_ in our study (r = 0.768, p < 0.001), and this correlation has been previously reported [28, 29]. 6MWD may be the most useful prognostic factor since it indirectly represents the lung diffusion capacity.

Among patients with IPF, an independent prognostic factor was low BMI, not 6MWD. This result is partly because pulmonary function and exercise tolerance were maintained relatively well in patients with IPF. Indeed only 3 patients (9%) had a 6MWD less than 250 m (Table 2). Nevertheless, the mortality of IPF patients was similar to that of patients with unclassifiable ILD and other ILDs. Thus, patients with IPF may need to be registered on the waiting list for transplantation earlier than those with other types of ILD. Low BMI was reported to be associated with shorter survival in US and Asian IPF cohorts [30, 31], although the cut-off level of BMI differed between studies. Nadrous et al. investigated the outcome in IPF patients younger than 50 years, and found that these patients had a similarly poor prognosis as older patients [32]. However, no prognostic factors had been identified in younger patients with IPF. Low BMI may be a unique prognostic factor in young and advanced patients with IPF.

Like the US ILD and IIP candidates listed for lung transplantation in previous studies, the most frequent diagnosis in our study was IPF. However, our study participants were younger and had a lower BMI than those in earlier studies, mainly due to the different ethnicity and upper age limit for transplant candidates [20, 33]. Therefore, the Japanese candidates for lung transplant may have specific clinical characteristics and prognostic factors which differ from the US and European cohorts, although 6MWD is one of the previously reported prognostic factors for IPF and ILD transplant candidates [19, 20].

In this study, we discovered intriguing characteristics present in young patients with advanced ILD. First, approximately 20% of candidates had unclassifiable ILD and approximately 40% with IPF had an “inconsistent with UIP pattern” by HRCT. Young patients with IIP may have a lower frequency of typical UIP pattern than elderly patients, by both HRCT and histology. Second, approximately 40% of patients with IPF had a family history of ILD. A previous report showed that patients with familial IIP, who showed a histological UIP pattern, had shorter survival times and younger average age at death [34]. Patients with familial IIP may frequently be included in the group of young and advanced patients with IPF.

There are some limitations of our study. First, the sample size was small. Although the present study was a single-center study, our hospital is the highest volume center for lung transplantation in Japan and one of the institutes that specialize in the care of ILD. Second, there were no longitudinal physiologic and laboratory data in our study. However, it was difficult to collect longitudinal data because most of the patients had severe respiratory impairments and poor outcomes.

In conclusion, our study demonstrated that Japanese patients with ILD on the waiting list for transplantation had very poor outcomes regardless of their specific diagnosis, and that 6MWD and BMI were independent predictors of mortality in patients with ILD and IPF, respectively. We may need to establish a rule to allocate the limited donor lungs based on prognostic factors, similar to the lung allocation system in the US, to save more patients with ILD.

## Supporting information

S1 Table Fine and Gray subdistribution hazards model (treating transplantation as a competing risk) results for evaluating the risk of mortality in lung transplant candidates with interstitial lung disease (n = 77). (DOCX)

S2 Table Fine and Gray subdistribution hazards model (treating transplantation as a competing risk) for evaluating the risk of mortality in patients with idiopathic pulmonary fibrosis (n = 33). (DOCX)
