# Supplementary material for: Prognostic factors and outcomes in Japanese lung transplant candidates with interstitial lung disease
Source: PLoS One. 2017 Aug 11;12(8):e0183171. doi: 10.1371/journal.pone.0183171 (PMC5553985; doi:10.1371/journal.pone.0183171)
Supplement: S1 Table — (DOCX) [file pone.0183171.s001.docx]

**S1 Table.** Fine and Gray subdistribution hazards model (treating transplantation as a competing risk) results for evaluating the risk of mortality in lung transplant candidates with interstitial lung disease (n = 77)

| *Univariate analysis* | Hazards ratio | 95%CI | | | p-value |
| --- | --- | --- | --- | --- | --- |
| Age, years | 1.01 | 0.98 | – | 1.04 | 0.64 |
| Male gender | 0.64 | 0.38 | – | 1.10 | 0.11 |
| IPF diagnosis | 0.63 | 0.35 | – | 1.13 | 0.12 |
| BMI, kg/m^2^ | 0.92 | 0.87 | – | 0.96 | < 0.01 |
| Ever smoker | 0.78 | 0.45 | – | 1.36 | 0.39 |
| Pulmonary hypertension | 1.07 | 0.55 | – | 2.07 | 0.85 |
| History of acute exacerbation | 1.48 | 0.74 | – | 2.98 | 0.27 |
| History of pneumothorax | 1.19 | 0.91 | – | 1.56 | 0.20 |
| %FVC, per 10%* | 0.76 | 0.62 | – | 0.93 | < 0.01 |
| %DL_CO_, per 10%† | 0.55 | 0.33 | – | 0.92 | 0.02 |
| 6MWD, per 10m | 0.97 | 0.95 | – | 0.99 | < 0.01 |
| Oxygen flow ≥2L/min at 6MWT | 2.20 | 1.23 | – | 3.94 | < 0.01 |
| GAP stage III | 2.77 | 1.23 | – | 6.23 | 0.01 |
| *Multivariate analysis* | Hazards ratio | 95%CI | | | p-value |
| *Model 1* |  |  |  |  |  |
| BMI, kg/m^2^ | 0.95 | 0.89 | – | 1.00 | 0.05 |
| %FVC, per 10%* | – |  | – |  | – |
| 6MWD, per 10m | 0.97 | 0.95 | – | 0.99 | < 0.01 |
| Oxygen flow ≥2L/min at 6MWT | – |  | – |  | – |
| *Model 2* |  |  |  |  |  |
| BMI, kg/m^2^ | 0.95 | 0.89 | – | 1.00 | 0.05 |
| 6MWD, per 10m | 0.97 | 0.95 | – | 0.99 | < 0.01 |
| Oxygen flow ≥2L/min at 6MWT | – |  | – |  | – |
| GAP stage III | – |  | – |  | – |

CI, confidence interval; IPF, idiopathic pulmonary fibrosis; BMI, body mass index; ILD, interstitial lung disease; %FVC, percent predicted forced vital capacity; %DL_CO_, percent predicted diffusing capacity of the lung for carbon monoxide; 6MWD, 6-min walking distance; 6MWT, the 6-min walk test; GAP stage, gender-age-physiology stage. *n = 71, †n = 50.
