# Supplementary material for: Prognostic factors and outcomes in Japanese lung transplant candidates with interstitial lung disease
Source: PLoS One. 2017 Aug 11;12(8):e0183171. doi: 10.1371/journal.pone.0183171 (PMC5553985; doi:10.1371/journal.pone.0183171)
Supplement: S2 Table — (DOCX) [file pone.0183171.s002.docx]

**S2 Table.** Fine and Gray subdistribution hazards model (treating transplantation as a competing risk) for evaluating the risk of mortality in patients with idiopathic pulmonary fibrosis (n = 33)

| *Univariate analysis* | Hazards ratio | 95%CI | | | p-value |
| --- | --- | --- | --- | --- | --- |
| Age, years | 1.001 | 0.96 | – | 1.06 | 0.78 |
| Male gender | 0.40 | 0.13 | – | 1.17 | 0.09 |
| BMI, kg/m^2^ | 0.83 | 0.74 | – | 0.93 | < 0.01 |
| Ever smoker | 0.66 | 0.26 | – | 1.64 | 0.37 |
| Pulmonary hypertension | 0.67 | 0.15 | – | 2.93 | 0.60 |
| History of acute exacerbation | 2.72 | 0.65 | – | 11.36 | 0.17 |
| History of pneumothorax | 2.19 | 0.81 | – | 5.92 | 0.12 |
| %FVC, per 10%* | 0.81 | 0.60 | – | 1.10 | 0.17 |
| %DL_CO_, per 10%† | 0.48 | 0.22 | – | 1.05 | 0.07 |
| 6MWD, per 10m | 0.96 | 0.93 | – | 0.995 | 0.03 |
| Oxygen flow ≥2L/min at 6MWT | 1.41 | 0.56 | – | 3.57 | 0.47 |
| GAP stage II or III | 1.68 | 0.62 | – | 4.53 | 0.31 |
| *Multivariate analysis* | Hazards ratio | 95%CI | | | p-value |
| Male gender | 0.34 | 0.13 | – | 0.93 | 0.04 |
| BMI, kg/m^2^ | 0.83 | 0.75 | – | 0.91 | < 0.01 |
| 6MWD, m | – |  | – |  | – |

CI, confidence interval; BMI, body mass index; ILD, interstitial lung disease;

%FVC, percent predicted forced vital capacity; %DL_CO_, percent predicted diffusing capacity of the lung for carbon monoxide; 6MWD, 6-min walking distance; 6MWT, the 6-min walk test; GAP stage, gender-age-physiology stage. *n = 32, †n = 28.
